# Parotid Gland Biopsy as an Additional Diagnostic Tool for Supporting the Diagnosis of Sjögren's Syndrome

**DOI:** 10.1155/2011/302527

**Published:** 2011-08-07

**Authors:** Muhammad S. Soyfoo, Xavier Catteau, Christine Delporte

**Affiliations:** ^1^Department of Rheumatology, Erasme Hospital, 808 Route de Lennik, 1070 Brussels, Belgium; ^2^Department of Anatomopathology, Erasme Hospital, 808 Route de Lennik, 1070 Brussels, Belgium; ^3^Laboratory of Biological Chemistry and Nutrition, Faculty of Medicine, Université Libre de Bruxelles, 808 Route de Lennik, 1070 Brussels, Belgium

## Abstract

Sjögren's syndrome (SS) is an autoimmune disease characterized by keratoconjunctivitis sicca and xerostomia. There are actually no diagnostic criteria for SS, but classification criteria based on the revised American-European criteria have been elaborated. These include subjective criteria: ocular and oral symptoms, and objective criteria: ocular, histopathological, oral, and serological signs. SS is considered if 4 of the 6 criteria are present, when histopathology or serology is positive, or if 3 of any 4 objective criteria are present. A patient presented with both ocular and oral symptoms and signs but did not meet the SS classification criteria. Indeed, no anti-SSA or anti-SSB antibodies were detected, and minor salivary gland biopsy was normal. To further understand the origin of the sicca symptoms, a parotid gland biopsy was performed and showed important lymphocytic infiltrates. This could account for the sicca symptoms and signs since parotid glands are one the major contributors to salivary flow. Therefore, parotid gland biopsy could be a useful asset for the diagnosis of SS.

## 1. Introduction

Sjögren's syndrome (SS) is an autoimmune disease with variable reported prevalence ranging from 0.1% to 4.8% [1, 2]. One of the hallmarks of this disease is the lymphocytic infiltration of both salivary and lacrimal glands responsible for keratoconjunctivitis sicca and xerostomia. Even though the main features of SS are sicca symptoms, the clinical spectrum of SS is broader and encompasses systemic signs and symptoms. SS can be classified as either primary or secondary associated with other autoimmune diseases such as systemic lupus erythematosus or rheumatoid arthritis. The pathogenesis of SS still remains to be fully understood although genetic and environmental factors might be involved [3].

There are presently no diagnostic criteria for SS, even though classification criteria based on the revised American-European criteria for SS have been made [4]. These criteria comprise subjective criteria: ocular symptoms and oral symptoms, and objective criteria: ocular signs, histopathological signs (focus  score ≥ 1), oral signs, and serological signs (presence of antinuclear antibodies, anti-SSA or anti-SSB). Patients are classified as SS if 4 of the 6 mentioned criteria are present, as long as histopathology or serology is positive, or if 3 of any 4 objective criteria are present.

From clinician's perspectives, diagnostic dilemma exists concerning a subgroup of patients presenting with severe sicca symptoms with the absence of antinuclear antibodies and the presence of a normal minor salivary gland biopsy. Since these patients did not meet the revised American-European classification criteria for SS, they are then classified as non-SS sicca syndrome. Repeating minor salivary gland biopsies is not recommended for the diagnosis of SS [5]. Normal labial salivary gland biopsies could then preclude the diagnosis of SS. Since parotid glands, among all salivary glands, contribute the most to salivary flow under stimulated conditions (the submandibular glands being the major contributor to salivary flow under unstimulated conditions and at night), severe damage of parotid glands could likely account for decreased salivary flow [6]. Consequently, parotid gland biopsy could be valuable for the diagnosis of SS in this subgroup of patients.

We hereby report a unique case of a patient associated with a high index of suspicion for SS due to severe sicca symptoms and signs, but presenting with a normal minor salivary gland biopsy and the absence of specific autoantibodies against SSA and/or SSB. Parotid gland biopsy, however, revealed important inflammation with a focus score of 3.

## 2. Case Report

**Figure 1:**
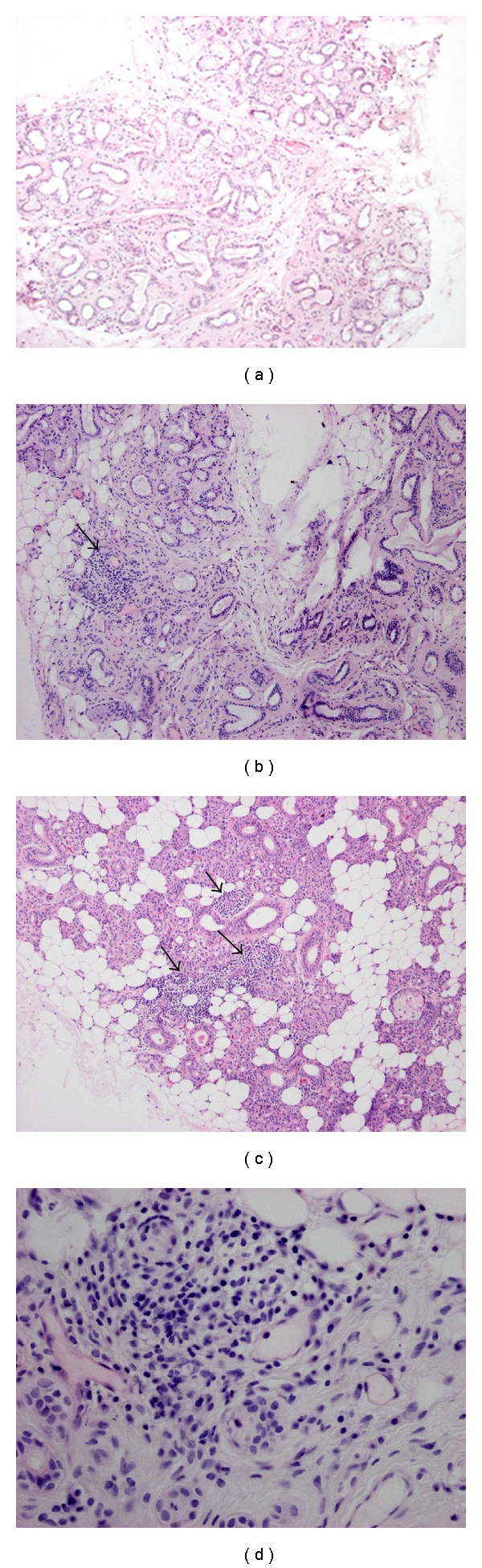
Histological analysis of salivary gland biopsies. Minor salivary glands ((a) first biopsy; (b) second biopsy) and parotid gland (c) biopsies were stained using hematoxylin-eosin. Original magnification: 100x. Arrows are pointing to lymphocytic infiltrates. (d) shows higher magnification (400X) of parotid gland biopsy depicting lymphocytic infiltrates.

A 54-year-old woman presented with dry mouth and eyes and arthralgia and was diagnosed as having fibromyalgia due to her 10-year history of complaints of these symptoms. Her past medical history includes total thyroidectomy for multinodular goitre and osteoporosis. Her current medications are L-thyroxine, alendronate, and nonsteroid anti-inflammatory agents. The patient did not smoke and had no alcoholic addiction. Due to the ocular and oral symptoms, the patient was suspected to have SS. Oral and ocular signs for SS were objectivised by the presence of a significant decrease in salivary flow (1.0 mL/15 min), a positive Schirmer's test (0.5 mm/5 minutes), a positive fluorescein-staining test (break-up time: 3 seconds), and a modified Van Bijsterveld score of 3, while salivary scintigraphy presented no abnormalities. To investigate the histopathological criteria for SS, a minor salivary gland biopsy was performed (lower lip biopsy with excision of 4 small lobules of labial salivary gland tissue with total surface area of 20 mm^2^) and did not depict focal lymphocytic infiltration (Figure 1(a)). Finally, the presence of autoantibodies, another objective criterion for SS, was evaluated and revealed positive antinuclear antibodies that were not identified as anti-SSA and/or anti-SSB antibodies (titer: 1 : 160). Additionally, complete blood sample analysis revealed normal C-reactive protein (CRP) levels, erythrocyte sedimentation rate, immunoglobulins, normal complement C4 level, and no hypergammaglobulinemia and rheumatoid factor. Other confounding factors such as viral infections (HIV, hepatitis C, HTLV-1), amyloidosis, sarcoidosis, and malignancy were excluded. Based on these results and according to the revised American-European classification criteria for SS, the patient was considered as having non-SS sicca symptoms. The patient was then treated with medications alleviating xerostomia and keratoconjunctivitis sicca: local ophthalmic drops and 5 mg of pilocarpine three times a day.

Twenty-four months later, the patient presented worsened sicca symptoms. Clinical examination of the patient revealed some pain when palpating the right parotid gland. An ultrasound examination of the right parotid gland showed a slightly enlarged gland, but the absence of canalicular stone. A second minor salivary gland biopsy was performed and showed a focus  score < 1 (Figure 1(b)). A Parotid gland biopsy was also done (performed under local anesthesia, with surgical excision and biopsy of 4 lobules of parotid gland tissue according to the technique by Kraaijenhaigen [7]) and displayed severe inflammatory infiltrates compatible with a grade 4 according to Chisholm's classification or a focus score of 3 but no lymphoepithelial lesions were seen (Figures 1(c) and 1(d)). Blood test analysis showed normal CRP levels, erythrocyte sedimentation rates, absence of hypergammaglobulinemia, normal levels of immunoglobulins, and the presence of nonspecific antinuclear antibodies distinct from anti-SSA or anti-SSB (titer: 1 : 80).

## 3. Discussion

According to the defined revised American-European classification criteria for SS, SS is considered when a patient meets 4 of the 6 criteria, as long as histopathology or serology is positive, or if 3 of any 4 objective criteria are met [4]. A patient was diagnosed as a nonsicca syndrome as the histopathology and serology criteria were not met. This case highlights one of the current diagnostic problems faced by physicians when confronted with patients with sicca symptoms, unspecific antinuclear antibodies, and normal or focus  score < 1 for minor salivary gland biopsies. As such, there are currently no diagnostic criteria for SS, and diagnosis of SS can be made according to the clinical insights of the physician. Even if substantial progress has been made in understanding the pathogenesis of SS, the absence of specific SS marker thereby undermines perspectives of diagnosis in a certain subgroup of patients with sicca symptoms. Using a limited cohort of patients, labial and parotid gland biopsies have been shown to display equivalent diagnosis potential in the diagnosis of SS [8, 9]. Another prospective study by Wise and colleagues did not support parotid biopsy as compared to minor labial salivary glands for establishing the diagnosis of SS [9]. However, due to its major contribution to salivary flow under stimulated conditions (while submandibular glands are the main contributors to salivary flow under unstimulated conditions and at night), parotid gland biopsy might yield better outcome in the diagnosis of SS than minor salivary gland biopsy [6]. There are also several lines of evidence supporting that both submandibular and parotid glands are affected in SS and that parotid saliva composition (high level of sodium) is altered in SS implying pathophysiological aberrations occurring at the cellular and glandular levels [10–12]. Besides, one main advantage of parotid biopsy relies on the fact that the parotid gland can be biopsied more often and is, therefore, not only a valuable diagnostic asset but also fundamental in monitoring disease treatment at the glandular level. Furthermore, benign lymphoepithelial lesions of parotid glands could represent a supplementary criterion for diagnosis of SS due to their absence in minor labial salivary gland biopsies [8].  It has to be underlined that in spite of having some advantages, performing a parotid biopsy can strike a heavy toll in terms of developing sialocelès, facial nerve damage, and Frey syndrome, if not done by experienced surgeons. Some patients might develop preauricular hypothesia, but, it is usually temporary [8]. Furthermore, in SS, the salivary gland tissue is replaced by fatty tissue, and risk of harvesting of fatty tissue is thereby increased if done by inexperienced physicians. In the study by Pijpe and colleagues, however, there was less morbidity due to parotid biopsy as compared to labial salivary gland biopsy, whereby a permanent sensory loss of 6% due to mental nerve damage was observed in patients undergoing minor salivary gland biopsy [8]. 

In conclusion, there are presently no diagnostic criteria for SS, and the diagnosis of SS is based upon clinical evidence of sicca syndrome, autoimmune markers, and histopathological evidence of salivary gland involvement. In those cases, where labial salivary gland biopsies are not conclusive and in the absence of specific autoantibodies such as anti-SSA and/or anti-SSB, parotid gland biopsy might be a supplementary diagnostic tool to support diagnosis. To lend futher support to this hypothesis, larger studies need to be performed to evaluate the diagnostic performance of parotid gland biopsy as an additional diagnostic tool in the evaluation of SS.

##  Conflict of Interests

The authors declare that they have no conflict of interests.
